# West Texas District Medical Society

**Published:** 1891-03

**Authors:** James Kennedy

**Affiliations:** Secretary


					﻿Western Texas Medical Association. — The Western
Texas Medical Association met in regular quarterly session on
the 28th inst., in San Antonio, with President Spring in the
chair.
Morning, evening and afternoon sessions were held, all of
which were well attended. The Committee on Necrology re-
ported suitable resolutions on the death of our lamented brother,
Dr. S. T. Lowry.
Dr. N. B. Field, of San Antonio, read a paper on “Premature
Labor,” in which he ably reviewed the history of this practice
and gave valuable hints as to the methods and indications of its
induction.
Dr. James Kennedy, of San Antonio, read a paper entitled
“Heredity as a Factor in Renal Pathology,” in which he en-
deavored to show the influence of heredity in causing disease of
the kidneys in an infant upon whom he held a post mortem, the
father of which child had died of suppurative nephritis.
Dr. A. Garwood, of New Braunfels, read a very able and
interesting essay upon “Carcinoma of the Stomach.”* The
thanks of the Association were tendered the authors for their
very able and instructive essays.
The subject of Aetiology of Pueumonia was broached by Dr.
VanGasken, of Luling, and was freely discussed by the members
present.
Small-pox and vaccination came in for a liberal share of dis-
cussion, but nothing new or original was developed.
An amendment to the by-laws was passed, compelling mem-
bers to pay dues under pain of expulsion.
The names of Drs. J. Leslie Smith, L. L. Lacy and B. J.
Norvierski were reported upon favorably by the Board of Censors
and were thereupon unanimously declared members of the Asso-
ciation.
It was decided upon to hold the next quarterly meeting in San
Antonio, whereupon the meeting adjourned.
James Kennedy, Secretary.
*Published in this number of the Journal.—Ed.
				

